# Estimate the contribution of incubation parameters influence egg hatchability using multiple linear regression analysis

**DOI:** 10.14202/vetworld.2016.806-810

**Published:** 2016-08-04

**Authors:** Mohamed H. Khalil, Mostafa K. Shebl, Mohamed A. Kosba, Karim El-Sabrout, Nesma Zaki

**Affiliations:** Department of Poultry Production, Faculty of Agriculture, Aflaton St., El-Shatby, University of Alexandria, Alexandria, Egypt

**Keywords:** chickens, hatchability, multiple regression, path coefficient, prediction

## Abstract

**Aim:**

This research was conducted to determine the most affecting parameters on hatchability of indigenous and improved local chickens’ eggs.

**Materials and Methods:**

Five parameters were studied (fertility, early and late embryonic mortalities, shape index, egg weight, and egg weight loss) on four strains, namely Fayoumi, Alexandria, Matrouh, and Montazah. Multiple linear regression was performed on the studied parameters to determine the most influencing one on hatchability.

**Results:**

The results showed significant differences in commercial and scientific hatchability among strains. Alexandria strain has the highest significant commercial hatchability (80.70%). Regarding the studied strains, highly significant differences in hatching chick weight among strains were observed. Using multiple linear regression analysis, fertility made the greatest percent contribution (71.31%) to hatchability, and the lowest percent contributions were made by shape index and egg weight loss.

**Conclusion:**

A prediction of hatchability using multiple regression analysis could be a good tool to improve hatchability percentage in chickens.

## Introduction

Poultry production is a process which has an important role in bridging shortage of nutrition in developing countries. The hatchability of chicken eggs is one of the most important parameters for efficiency in hatchery practices. Heritability estimated for hatchability in chickens was low [1]. Numerous parameters have pronounced influence on the hatchability of chicken eggs and it is necessary to know the important parameters affecting hatchability success and its contribution.

In this study, we chose five incubation parameters which have a direct effect on hatchability. Statistical models can be used in prediction and helps to make the best decisions to approach maximum productivity in the poultry industry. Path coefficient analysis becomes successful tool which can be used in examining the possible causal linkage between statistical variables and for prediction [2].

The main objectives of this study were: (1) To determine the most studied incubation parameters affecting hatchability percentage; (2) to calculate the path coefficients of hatchability on the studied parameters; (3) to calculate the multiple regression coefficients of hatchability on the studied parameters. This study is very important to reach optimum hatchability percent of chicken eggs. Furthermore, the prediction of hatchability percent using multiple regression analysis is necessary to improve hatchability percentage in chickens.

## Materials and Methods

### Ethical approval

The approval from the Institutional Animal Ethics Committee to carry out this study was not required as no invasive technique was used.

### Study design

This study was conducted at the Poultry Research Center, Alexandria University, Egypt. Four Egyptian chicken strains were used:

1. Alexandria (Alex): Obtained in 1958 by crossing between four strains of chickens (Fayoumi × Plymouth Rock × Rhode Island Red × White Leghorn) [3]
2. Fayoumi (Fay): Local Egyptian chicken strain
3. Matrouh (Mat): Obtained in 1974 by crossing between Doki-4 and white Leghorn [4]
4. Golden Montazah (G.M): Obtained in 1974 by crossing between Doki-4 and Rhode Island Red [5].

A total of 16 individual breeding pens have been used to produce chickens of the four strains (Alex, Fay, Mat, and G.M) used in this study. In each pen had one sire mated to 12 dams, with 4 replicates for each. Age of stock was 50 weeks. The experimental parents and hatching eggs exposed the same managerial treatments for all strains. The identified eggs were pedigreed for each dam through trap nesting. After that, the collected eggs were weighed and determined the egg shape index before set in an electric forced draft incubator. A total of 3144 eggs (Alex: 912, Fay: 683, Mat: 840, and G.M: 709) were collected in five hatches and taken during the period from December to January. During hatching, eggs were weighed at the 7^th^ and 18^th^ day of incubation using an electric digital scale to calculate the egg weight loss. On the 7^th^ and 18^th^ day of incubation, the eggs were candled and those with evidence of living embryos were transferred to the hatcher in individual pedigree boxes. On day 21, the hatched chicks were weighed and percentages of hatchability were calculated.

Hatchability percentage (Commercial)

- Hatchability of total eggs:
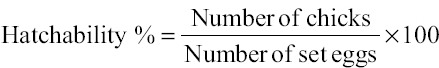
- Hatchability of fertile eggs (Scientific)
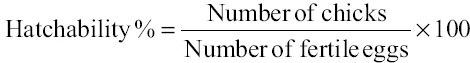

Hatching chick weight: Individual chick body weight (g) at hatching time.

### Statistical analysis

Data were analyzed using IBM SPSS Statistics for Windows, version 20.0 [6].

- Multiple linear regression was performed on the studied parameters to determine the most influencing parameters on hatchability. The model for the multiple linear regression was as follows:
  Y = a + b_1_X_1_+ b_2_X_2_+…… +b_6_X_6_
  Where,
  Y = Response variable (hatchability %),
  a = Intercept,
  b = Partial regression coefficient,
  X = Independent variables (shape index, egg weight, early dead, late dead, egg weight loss, and fertility).
- Path coefficient: Standardized partial of regression coefficients were calculated. It was to involve a direct comparison of values to reflect the importance relative of independent variables (X) to explain variation in the dependent variable (Y). The path coefficient from an explanatory variable (X) to a response variable (Y) as described by Mendes *et al*. [7] is shown below:
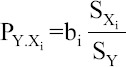
  Where,
  P_YXi_ = Path coefficient from X_i_ to Y (i = parameters affecting),
  b_i_ = Partial regression coefficient,
  = Standard deviation of X_i_,
  S_Y_ = Standard deviation of Y.
- The significance of each path coefficient in the multiple linear regression model was tested by t-test.
- Coefficient of determination (R^2^) was calculated as follows:
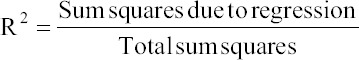

## Results and Discussion

Regarding the studied strains (Table-1), significant differences in commercial and scientific hatchability among strains were observed. However, Alexandria strain has the highest significant (p≤0.01) commercial hatchability (80.70%), over that for Matrouh (64.68%), Fayoumi (57.41%), and Montazah (45.26%) strains. In respect of scientific hatchability percentages, Alexandria, Montazah and Fayoumi strains have statistically equal significant (p≤0.05) values (92.59, 87.69 and 87.57%, respectively), over that for Matrouh strain (70.69%).

**Table-1:** Means and standard error (X̄^1^±SE^2^) of hatchability (%) by strain along with test of significance.

Strains	N	Commercial hatchability (%)		Scientific hatchability (%)	
	
		X̄^1^	SE^2^	X̄^1^	SE^2^
Alexandria	11	80.70^A^	2.78	92.59^A^	3.57
Fayoumi	12	57.41^C^	3.42	87.57^A^	4.43
Matrouh	6	64.68^B^	5.11	70.69^B^	5.17
Montazah	12	45.26^D^	5.16	87.69^A^	5.71
Factor		p value	Significant	p value	Significant
Strains		0.002	**	0.048	*

1 Means of actual data,

2 SE for transformed data.

* p≤0.05;

** p≤0.01, Means having different letter for each character are significantly different (p≤0.05). SE: Standard error

According to the indicative performance records for scientific hatchability (>93% excellent, >90% very good, >87% good, >83% average, and <83% poor) [8], the assessment of the percentages of this parameter obtained here in this study (Table-1) according to the previous categories indicated that Alexandria strain has very good hatchability, Fayoumi, and Montazah have good hatchability, while Matrouh has poor hatchability. The significant hatchability between strains may be due to fertility.

Alsobayel and Albadry [9] reported that hatchability percentages were significantly affected by breed. Allanah *et al*. [10] revealed that hatchability was the highest in the improved and native chicken strains, followed by the exotic strains which recorded the least percentage hatchability.

In addition, highly significant differences in hatching chick weight among strains were observed. Alexandria, Montazah, and Matrouh strains have statistically equal significant hatching chick weight (37.54, 37.78, and 36.64 g, respectively), while Fayoumi was 33.11 g. The significant difference among strains may be due to the significant difference in egg weight.

### Establishment of preliminary and optimized multiple regression equations

#### Preliminary multiple regression equation

**Figure-1:**
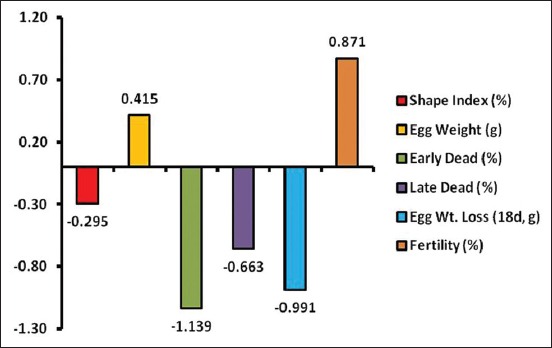
Multiple regression of hatchability on the studied parameters.

Multiple linear regression analysis gives the amount by which the dependent variable (hatchability %) increases when one independent variable (studied parameters) is changed by one unit and all the other independent variables are held constant. In particular, the value of the partial coefficient for one independent variable will vary, depending on the other independent variables included in the regression equation. Multiple regression equation of hatchability (%) on the parameters studied was as follows (Table-2 and Figure-1).

**Table-2:** Multiple regression, path coefficients, and its contribution of commercial hatchability (%) on the studied parameters.

Commercial hatchability (%)	Regression coefficient	Path coefficient	Contribution (%)	p value	Significant
(Constant)	55.42				
Shape index (%)	−0.295	−0.023	1.76	0.257	NS
Egg weight (g)	0.415	0.069	5.28	0.001	***
Early dead (%)	−1.139	−0.157	12.01	0.000	****
Late dead (%)	−0.663	−0.085	6.5	0.000	****
Egg weight loss (18 d, g)	−0.991	−0.041	3.14	0.053	NS
Fertility (%)	0.871	0.932	71.31	0.000	****
R^2^	0.86				

*** p≤0.001,

**** p≤0.0001. NS=Not significant

Hatchability = 55.42 − 0.29 SI + 0.42 EW − 1.14 ED − 0.66 LD − 0.99 EL + 0.87 F

Where, SI = Shape index, EW = Egg weight, ED = Early dead, LD = Late dead, EL = Egg wt. loss, and F = Fertility.

Partial regression coefficients are the slope coefficients (b’s) in a multiple regression model. The partial regression coefficients were −0.29, 0.42, −1.14, −0.66, −0.99, and 0.87% for these independent variables (SI, EW, ED, LD, EL, and F, respectively) (Table-2).

This equation implies that one unit decrease in these parameters (SI, ED, LD, and EL) will increase hatchability by 0.29%, 1.14%, 0.66%, and 0.99%, respectively, within the minimum and maximum values of these parameters, and all the other independent variables are held constant. On the other hand, the independent variables (EW and F) increase with one unit, hatchability will increase by 0.42% and 0.87%, respectively, within the minimum and maximum values of these parameters; all the other independent variables are held constant (Table-2). The independent variables (EW, ED, LD, and F) had a significant effect on hatchability. However, the rest independent variables studied (SI and EL) were not significant.

Egg weight loss did not significantly affect hatchability. This may due to the water lost from eggs was within the normal lost during incubation. Egg weight loss ranged from 10.03% to 10.96% by strain in this study. Ha [11] cleared that the best hatchability is obtained with poultry species when eggs lose 12% of their fresh weight from time of lay to time the embryo opens the shell, and hatchability decreases for eggs losing <10% or >15% of their fresh egg weight. In addition, this may be attributed to reflection of functional porosity of the shell and the initial mass of each egg. Abiola *et al*. [12] reported that egg weight loss may affect hatchability through the proportion of pore areas and pore diameter regardless of the size of egg.

#### Path coefficients

The path analysis has as intermediary calculation the normal equation system such as multiple regression. Path coefficients of hatchability on the independent parameters studied are presented in Table-2. The equation of path coefficient implies that one unit decrease in standard deviation of SI, ED, LD, EL results in an increase of hatchability equal to 0.023, 0.157, 0.085 and 0.041, respectively. However, EW and F increase with one unit; hatchability will increase by 0.069 and 0.932, respectively.

#### Contribution percentage of studied parameters

**Figure-2:**
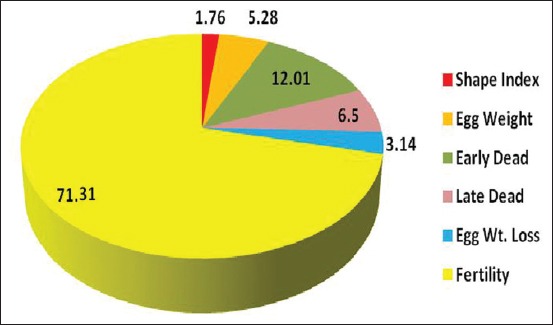
The contribution of the studied parameters on hatchability.

Fertility made the greatest percent contribution (71.31%) to hatchability in chickens (Table-2), followed by early embryo dead by 12.01%, late embryo dead by 6.50% and egg weight by 5.28%. The lowest percent contributions were made by egg weight loss and shape index by 3.14% and 1.76%, respectively (Table-2 and Figure-2).

#### Optimized multiple regression equation

Because the partial regression coefficients of some characters studied (shape index and egg weight loss) were statistically not significant, thus these characters were expunged from the regression model to obtain a much more simplified equation. After the removal of the redundant variables from the initial regression equations, the optimized but much simplified equation model (Table-3) with their coefficient of determination was:

**Table-3:** Multiple regression, path coefficients, and its contribution of commercial hatchability (%) on the significant studied parameters.

Parameters	Regression coefficient	Path coefficient	Contribution (%)	p value	Significant
(Constant)	18.678				
Egg weight (g)	0.336	0.056	4.55	0.007	**
Early dead (%)	−1.143	−0.158	12.84	0.000	****
Late dead (%)	−0.679	−0.087	7.07	0.000	****
Fertility (%)	0.869	0.930	75.55	0.000	****
R^2^	0.86				

** p≤0.01,

**** p≤0.0001

Hatchability = 18.67 + 0.34 EW − 1.14 ED − 0.68 LD + 0.87 F

Where (EW = Egg weight, ED = Early dead, LD = Late dead, and F = Fertility).

**Figure-3:**
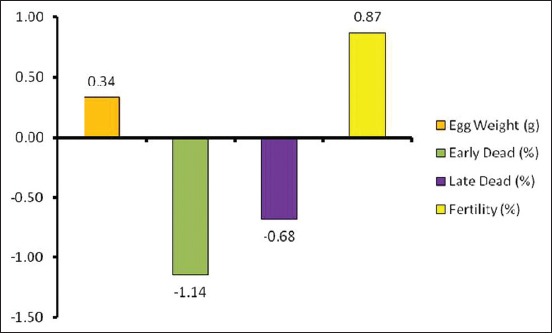
Multiple regression of hatchability on the significant studied parameters.

This equation implies that one unit increase in these parameters (EW and F) will increase hatchability by 0.34% and 0.87%, respectively, within the minimum and maximum values of these parameters; and all the other independent variables are held constant. On the other hand, ED and LD if decreased with one unit, hatchability will increase by 0.1.14% and 0.68%, respectively (Table-3 and Figure-3).

Similar results were reported by Wolanski *et al*. [13] and Alabi *et al*. [14] in chickens. They reported that egg weight was effective on hatchability of fertile eggs. The medium and large-sized eggs had significantly higher hatchability values than small-sized eggs. In addition, they found that medium-sized eggs had significantly higher hatchability than both small and large-sized eggs. Quadratic regression analysis revived that hatchability was optimized in eggs of Potchefstroom Koekoek chickens weighting approximately 51 g (r^2^=100). If performance (e.g., weight gain, live weight, feed intake, feed conversion ratio, mortality) is of high importance, large eggs can be considered [14]. Moreover, De Witt and Schwalbach [15] found that large-size eggs recorded to have higher hatchability percent in Rhode Island Red and New Hampshire chickens. In addition, working on three rural chickens’ breeds, Abdul Rashid *et al*. [16] found that large-sized eggs had higher hatchability than small sized eggs. Moreover, the categories of egg weight significantly affected mortality of embryos. The highest early and late embryonic mortalities were observed in small-size egg weight followed by that of medium and large-size egg weight. Higher mid embryonic mortality was observed in small and large egg weight categories than in medium ones [17].

On the other hand, Ramaphala [18] found no differences between different egg sizes were detected with hatchability percentage and percentage hatch of fertile in Cobb 500 broiler chickens breeder eggs.

The increase of early and late embryonic mortalities of eggs caused a reduction in hatchability. The findings of Elibol and Brake [19] supported these results. They indicated that fertile hatchability decreased due to increased percentage early and late embryonic mortalities of broiler eggs.

#### Path coefficients

The equation of path coefficient (Table-3) implies that one unit increase in standard deviation of EW and F results in an increase of hatchability equal to 0.056 and 0.93, respectively. However, ED and LD decrease with one unit in standard deviation, hatchability will increase by 0.158 and 0.087, respectively.

#### Contribution percentage of studied parameters

**Figure-4:**
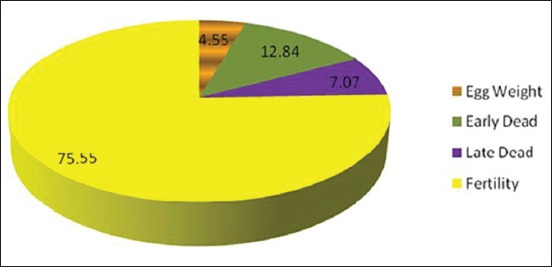
The contribution of significant studied parameters on hatchability.

Fertility made the greatest percent contribution (75.55%) to hatchability in chickens (Table-3), followed by early embryo dead by 12.84%, late embryo dead by 7.07%, and egg weight by 4.55% (Table-3 and Figure-4).

## Conclusion

The prediction of hatchability percent using multiple regression analysis could be a good method for estimating the performance and breeding value of chickens. Furthermore, the relationships existing between hatchability and other parameters studied may be useful in breeding plan, thereby providing a basis for the genetic manipulation and improvement of the native stock.

## Authors’ Contributions

MHK, MKS and MAK carried out the experiment design, statistical analysis and data interpretation. KE participated in practical work and wrote the manuscript. MHK and KE had the primary responsibility for the content of the manuscript. All authors read and approved this manuscript.
